# Osteonecrosis of the jaw in patients with clear cell renal cell carcinoma treated with targeted agents: a case series and large-scale pharmacovigilance analysis

**DOI:** 10.3389/fphar.2024.1309148

**Published:** 2024-10-29

**Authors:** Shuyun Wang, Rui Zhang, Song Wang, Qian Guo, Donghong Yin, Yan Song, Xianhua She, Xuyan Wang, Jinju Duan

**Affiliations:** ^1^ Department of Pharmacy, School of Pharmacy, Shanxi Medical University, Taiyuan, Shanxi, China; ^2^ Department of Pharmacy, Second Hospital of Shanxi Medical University, Taiyuan, Shanxi, China; ^3^ Central Laboratory, Shanxi Hospital of Integrated Traditional Chinese and Western Medicine, Taiyuan, Shanxi, China

**Keywords:** tyrosine kinase inhibitors, immune checkpoint inhibitors, osteonecrosis of jaw, FAERS, pharmacovigilance

## Abstract

**Objective:**

To optimize the use of tyrosine kinase inhibitors (TKIs) and immune checkpoint inhibitors (ICIs) for cancer patients, we characterized and evaluated ONJ related to TKIs and ICIs by analyzing a public database and reviewing the relevant literature. TKIs and ICIs are limited to drugs that treat renal cancer recommended by the National Comprehensive Cancer Network Clinical Practice Guidelines in Oncology for Kidney Cancer.

**Methods:**

We described a case series of patients experiencing ONJ while on TKIs or ICIs. We also analyzed spontaneous reports submitted to the FAERS in an observational and retrospective manner between January 2004 and December 2022. Selecting ONJ’ adverse events to TKIs and ICIs. Associations between TKIs, ICIs and ONJ were assessed using reporting odds ratios (ROR), drug interaction signals based on the Ω shrinkage measure.

**Results:**

29 patients with ONJ events while on TKIs and ICIs were included in our case series. 240 were related to ONJ AEs. Specifically, 32.1% ICSRs were linked to sunitinib, 16.7% to lenvatinib, 12.9% to pazopanib, 12.5% to nivolumab, 10.0% to axitinib, 5.4% to sorafenib, 5.0% to pembrolizumab, 4.2% to cabozantinib, and 1.3% to ipilimumab. More ICSRs were generally seen in male and reported in Europe. The median age was 63 years. Renal cancer and lung cancer was the most common indication for TKIs and ICIs, respectively. Excluding missing data, the prevalence of mortality was highest for sunitinib-related ONJ ICSRs (18.5%), followed by sorafenib-related ONJ ICSRs (15.4%). With the criteria of ROR, sunitinib and lenvatinib were significantly associated with ONJ AEs. With the criteria of Ω, nivolumab + cabozantinib was significantly associated with ONJ AEs.

**Conclusion:**

TKIs and ICIs have been reported to have significant ONJ side effects. Patients and physicians need to recognize and monitor these potentially fatal adverse events.

## 1 Introduction

Renal cell carcinoma (RCC) is responsible for approximately 3% of all cancers, with the highest incidence occurring in the West, and RCC has shown an annual growth rate of approximately 2% in the last two decades (Paragliola et al., 2023). In 2023, it is estimated that RCC will be the 6th and 9th most common malignancy in males and females, respectively, in the United States. It is also vital to consider that many patients with advanced RCC develop bone metastases in their disease. Indeed, recent research shows that up to 35% of patients are in this category, highlighting the need for careful monitoring and effective management of this potential complication. Bone involvement in RCC is frequently related to skeletal-related events (SREs), which can significantly impact the quality of life of patients and adversely affect their survival. Specifically, the prevention of SREs often involves systemic targeted therapies.

Osteonecrosis of the jaw (ONJ), a rare but potentially severe disease, may affect both jaws but is more common in the jaw. In particular, ONJ manifests as one or more necrotic bone lesions that persist for at least 8 weeks in patients who treated with antiresorptive medicines for primary or metastatic bone cancer, Paget’s disease, or osteoporosis, but with no history of jaw radiation therapy (Aghaloo et al., 2015). The first case of ONJ linked to bisphosphonates was reported in 2003, and the number of similar cases has since increased. The American Association of Oral and Maxillofacial Surgeons (AAOMS) updated the definition of “bisphosphonate osteonecrosis of the jaw” (Schwartz, 2015) in 2014 and 2015 and recommended a change in the nomenclature of “drug-associated osteonecrosis of the jaw”. Indeed, this nomenclature modification was considered necessary due to the increasing number of cases where mandible necrosis is associated with other antiresorptive (denosumab) or anti-angiogenic therapy (Paragliola et al., 2023). Notably, in the 1990s, advanced RCC was primarily treated with high doses of IL-2 and interferon-alpha (Tran and Ornstein, 2022), whereas in recent years, kinase inhibitors (TKIs) and immune checkpoint inhibitors (ICIs) have become available (Sánchez-Gastaldo et al., 2017). TKIs and ICIs, whether as monotherapy or combination therapy (Powles and ESMO Guidelines Committee, 2021), have significantly improved survival in patients with advanced RCC but often at the expense of adverse events (AEs) that can worsen the quality of life of the patient (Postow et al., 2018; Huang and Hsieh, 2020; Iannantuono et al., 2022). Previous studies have indicated that it is crucial for TKI- and ICI-induced AEs to be addressed, as the incidence of TKI-related AEs ranges from 87.2% to 100% and that of ICI-related adverse events ranges from 69% to 100% (De Britto Evangelista et al., 2022). Specifically, TKIs are known to cause albuminous-associated AEs, diarrhea, high blood pressure, and palmar-plantar erythrodysesthesia (Ancker et al., 2017; Belum et al., 2016), and ICIs affect a wide range of organs, particularly gastrointestinal and endocrine organs, the skin, liver, lungs, and joints. However, other significant side effects, such as ONJ, have not been well characterized in patients with renal cancer treated with TKIs and ICIs. The understanding of AEs is critical for TKIs and ICIs to be successful, as the interruption or reduction of the anti-cancer dose is harmful to the long-term efficacy of treatment (Bellmunt et al., 2011). In addition, a number of studies (Fleissig et al., 2012; Gianoukakis et al., 2018; Hu et al., 2019; Kim et al., 2018) have emphasized that the early management of AEs is crucial for the outcome of therapy (Ancker et al., 2019).

The Food and Drug Administration (FDA) Adverse Event Reporting System (FAERS), which collects information on all AEs for the FDA, is an essential tool for post-marketing safety monitoring in the United States. The FAERS data should be available to the public, and if potential safety concerns are identified, further assessments should be conducted to assess possible changes in product label information, limitations on the duration of use of the drug, or withdrawal of the medicine from the market (Egeberg and Thyssen, 2023).

Previously, no case series has evaluated more than 10 cases. Therefore, the timing, spectrum, and clinical manifestations of ONJ events due to systemic treatment options, as recommended by the National Comprehensive Cancer Network Clinical Practice Guidelines in Oncology for Kidney Cancer (Version 4.2023 - 18 January 2023) (Table 1), for patients with clear cell RCC are poorly understood. In this study, we reviewed the published literature and consulted FAERS on the incidence of ONJ following ICI and TKI treatment to provide a comprehensive description of drug-related ONJ and determine whether there are any safety signals between ONJ and systemic treatment (either monotherapy or a combined regimen). The results of this study will be helpful to optimize the use of therapeutic drugs for cancer.

**TABLE 1:** List of the main anticancer agents available for RCC treatment as monotherapy and/or combined regimens.

Drug	Targets
Axitinib	VEGFR 1-3, PDGFRβ
Cabozantinib	RET, MET, VEGFR1-3, KIT, TrkB, FLT-3, AXL, TIE-2, ROS1
Lenvatinib	VEGFRs, FGFRs, PDGFR, KIT, RET
Pazopanib	VEGFR1/2/3, PDGFRα/β, FGFR1/3, KIT, LCK,FMS, ITK
Sunitinib	PDGFRα/β, VEGFR1-3, KIT, FLT-3, CSF-1R, RET
Sorafenib	VEGFR-2 and -3, FLT-3, PDGFR β, c-KIT, RET, and RAF

Drug	Target
Avelumab	PD-L1
Ipilimumab	CTLA-4
Nivolumab	PD-1
Pembrolizumab	PD-1

Drugs	FDA Approval
Lenvatinib + Everolimus	2016
Nivolumab + Ipilimumab	2018
Pembrolizumab + Axitinib	2019
Avelumab + Axitinib	2019
Nivolumab + Cabozantinib	2021
Pembrolizumab + Lenvatinib	2021

Abbreviations: cytotoxic T-lymphocyte associated protein 4 (CTLA-4); colony-stimulating factor 1 receptor (CSF- 1R); fibroblast growth factor receptors (FGFR); Fms-like tyrosine kinase 3 (FLT3); IL2 inducible T cell kinase (ITK); lymphocyte-specific protein tyrosine kinase (LCK); programmed Cell death protein 1 (PD-1); platelet-derived growth factor receptor (PDGFR); programmed death-ligand 1 (PD-L1); endothelial-enriched tunica internaendothelial cell kinase 2 (TIE2); tropomyosin receptor kinase B (TrkB).

## 2 Materials and methods

### 2.1 Case series

We performed a search of the literature using PubMed and Embase, including articles written in English and other languages published between 2003 and 2023, with the following query: “bisphosphonates, osteonecrosis, jaw or jaws, cancer, RCC, immune checkpoint inhibitors, checkpoint inhibitor therapy, targeted therapy, checkpoint inhibitor treatment, ipilimumab, pembrolizumab, avelumab, nivoluma, sunitinib, sorafenib, axitinib, cabozantinib, pazopanib, lenvatinib.” Only studies with abstracts and articles citing renal or kidney cancer were selected and examined. Additional data have been obtained from the literature on renal carcinoma, including age, sex, time to onset, bisphosphonates, and tumors.

### 2.2 Phamacovigilance analysis

This observational and retrospective pharmacovigilance study utilized a disproportionality analysis according to the individual case safety reports (ICSRs) taken from FAERS (range from 2004 quarter 1 to 2022 quarter 4). The FAERS database is a freely accessible public database. This database includes anonymous reports from healthcare professionals, consumers, producers, and others. The FAERS dataset is made up of seven data tables, which contain patient demographic information (e.g., sex, age, weight), the source and type of the report, the country of the report, the drugs used with start and end dates (when available), the doses and routes of administration, AEs and their outcomes, and indications of use, and its data can be used to generate scientific publications. Therefore, we used this data to investigate the potential association between ONJ and TKIs, ONJ and ICIs for RCC. Ethical approval was not required because this study was conducted using de-identified data.

In the database, AEs were coded using the preferred terms (PTs) and System Organ Classes (SOCs) in the Medical Dictionary for Regulatory Activities (MedDRA).

### 2.3 Descriptive analysis

The following data were retrieved from FAERS for this study: the patient’s sex and age at the time of AE, the reporting year and country, the AE, as well as its occurrence date, seriousness criteria, and outcome. Furthermore, the following TKI and ICI variables were extracted: the name of the drug, the role of the drug in the occurrence of the event (suspected, concurrent, or interaction), and the date of prescription, if available.

One of the main problems with spontaneous reporting is the existence of duplicates (that is, the same report from different sources) and multiple reports (i.e., a follow-up of the same case with additional and updated information). In this study, we used two steps to remove duplicates. Firstly, only the most recent version of the cases for which monitoring was available was used. Secondly, cases with the same event, event date, age, gender, and country of origin were considered duplicates. Reports for which the reported start date of the drug was later than the AW date were considered anomalous and were excluded.

### 2.4 Statistical analyses and signal detection

A descriptive analysis of all the ICSRs was conducted to assess the demographic characteristics and variables related to the drugs under study. The analyses were performed considering the demographic data of ICSRs (gender and age), reporter country, indication, time to report, and AE outcome. The outcome of the AEs was classified as “Death,” “Death/Disability/Other,” “Disability,” “Disability/Congenital Anomaly/Other,” among others. In the case of two or more AEs with different results reported in a single ICSR, the result with the lowest resolution level was chosen for classification. The time to onset of ONJ events was determined based on the time elapsed between the onset of the AE and the start of therapeutic intervention.

Statistical analysis was performed to identify statistical associations between the drugs under study and all AEs. The reporting probability ratio (ROR) was used to identify signals suggesting a potential increased risk of drug-related AEs for the drugs being studied. A two-by-two contingency table was used to compute the ROR. If the lower 95% confidence interval was >1 and the reported count was ≥3, AEs was considered to be positive. A higher ROR indicates a stronger disproportion and intensity signal, suggesting that a particular drug is more likely to induce a particular adverse reaction than any other drug (Cirmi et al., 2020). A number of algorithms can also be used (Noguchi et al., 2019) to identify drug-drug interaction (DDI) signals. Of these, the measurement of Ω(Norén et al., 2008) used by Uppsala Monitoring Centre (UMC) (Xia et al., 2022) showed a more conservative detection tendency compared to a previous study (Noguchi et al., 2020). A cut-off value of 95% CI for Ω (Ω025) > 0 was used as the detection criterion in this work.

## 3 Results

### 3.1 Case series

Twenty-five cases of ONJ induced by TKIs and four cases of ONJ induced by ICIs (Table 2) were identified in the study. For the TKI-related ONJ cases, the median patient age was 63 years, the median time to onset was 12.8 months, 68.2% of patients were male (missing data were excluded), and the most common malignancy involved (65.64%) was renal cell carcinoma. Furthermore, sunitinib was implicated in 21 (84.0%) patients, lenvatinib was implicated in 2 (8.0%) patients, and cabozantinib and axitinib were each implicated in 1 (4.0%) patient. For the ICI-related ONJ cases, the median patient age was 53 years, the median time to onset was 10.2 months, two patients were male and two were female, and all cases had advanced melanoma. Ipilimumab (CTLA4 inhibitor) was implicated for two (50.0%) of the patients, nivolumab and pembrolizumab were implicated for one (25.0%) patient each, as shown in Table 2.

**TABLE 2:** Summary of case reports of immune checkpoint inhibitor-induced pure red cell anaemia reported in the literature.

	Age	Sex	Bisphosphonate	Malignancy	Targeted agent	Duration (month)
1 (Agrillo et al., 2012)	65	M	Yes	RCC	Sunitinib	13
2	67	M	Yes	RCC	Sunitinib	13
3 (Koch et al., 2010)	59	M	No	RCC	Sunitinib	12
4 (Fleissig et al., 2012)	58	F	No	RCC	Sunitinib	12
5 (Hoefert and Eufinger, 2010)	55	-	Yes	RCC	Sunitinib	9
6 (Hoefert and Eufinger, 2010)	56	-	Yes	RCC	Sunitinib	4
7 (Hoefert and Eufinger, 2010)	62	-	Yes	RCC	Sunitinib	23
8 (Brunello et al., 2009)	59	M	Yes	RCC	Sunitinib	4
9 (Nicolatou-Galitis et al., 2012)	19	F	No	RCC	Sunitinib	-
10 (Nicolatou-Galitis et al., 2012)	64	F	No	RCC	Sunitinib	48
11 (Bozas et al., 2010)	51	M	Yes	RCC	Sunitinib	22
12 (Melloni et al., 2015)	62	M	Yes	RCC	Sunitinib	18
13 (Soós et al., 2015)	42	F	Yes	Gastric Cancer	Sunitinib	-
14 (Moghaddas et al., 2017)	53	M	Yes	RCC	Sunitinib	5
15 (Marino et al., 2015)	51	F	No	MTC	Cabozantinib	3
16 (Abel Mahedi Mohamed et al., 2018)	70	M	No	RCC	Sunitinib	9
17 (Kuroshima et al., 2022)	58	M	No	Thyroid Cancer	Lenvatinib	12
18 (Monteiro et al., 2021)	61	F	No	Thyroid Cancer	Lenvatinib	-
19 (Mavroidis and Lind, 2009)	55	F	Yes	RCC	Sunitinib	16
20 (Patel et al., 2017)	67	M	No	RCC	Axitinib	2
21 (Christodoulou et al., 2009)	48	M	Yes	RCC	Sunitinib	3
22 (Fusco et al., 2015)	48	M	Yes	RCC	Sunitinib	14
23 (Fusco et al., 2015)	57	M	Yes	RCC	Sunitinib	7
24 (Fusco et al., 2015)	29	M	Yes	RCC	Sunitinib	4
25 (Vallina et al., 2019)	63	M	Yes	RCC	Sunitinib	30
26 (Guida et al., 2020)	58	F	No	Melanoma Cells	Ipilimumab	36
27 (Owosho et al., 2015)	52	M	No	Melanoma Cells	Ipilimumab	2.2
28 (Pundole et al., 2020)	75	M	No	Melanoma Cells	Nivolumab	1.5
29 (Decaux and Magremanne, 2020)	28	F	No	Melanoma Cells	Pembrolizumab	1

### 3.2 Demographic characteristics

From January 2004 to December 2022, the FAERS database contained 19, 494, 698 reports, of which 16, 134, 686 reports were retained after duplication records were excluded. Overall, there were 90,324 ICSRs (sunitinib: 21,840; cabozantinib: 4,324; lenvatinib: 13,802; axitinib: 12,697, pazopanib: 23,265; sorafenib: 14,396) suspected to be related to TKIs and 10,3123 ICSRs (pembrolizumab: 32,010; nivolumab: 57,291; avelumab: 1,560; ipilimumab: 12,262) suspected to be related to ICIs. Notably, 240 ICSRs were related to ONJ AEs. Specifically, 77 (32.08%) ICSRs were linked to sunitinib, 40 (16.67%) to lenvatinib, 31 (12.92%) to pazopanib, 30 (12.50%) to nivolumab, 24 (10.00%) to axitinib, 13 (5.42%) to sorafenib, 12 (5.00%) to pembrolizumab, 10 (4.17%) to cabozantinib, and 3 (1.25%) to ipilimumab.

In terms of the ONJ-related ICSRs for TKIs, the highest number of ICSRs occurred in 2022. Specifically, reports related to axitinib and cabozantinib were collected since 2014, and the highest number of ICSRs occurred in 2022. For pazopanib, sunitinib, and sorafenib, ONJ AE reports were collected since 2010, and the highest numbers of ICSRs occurred in 2015, 2013, and 2011, respectively. For lenvatinib, ONJ AEs were collected since 2016, and the highest number of ICSRs occurred in 2022. In general, most cases were reported from North America and Europe. The median age of patients from all the TKI-related ICSRs was 63 years (IQR, 56–70 years; n = 143). More than half of the ICSRs related to male patients (63.1%). It is interesting that renal cancer was the most common indication for all the TKIs (65.64%), followed by thyroid cancer (10.26%). For all TKI-related ONJ cases documented in ICSRs (excluding missing data), the most common adverse outcome was other serious (88/168, 52.38%), and the most serious outcome was death/life-threatening condition (22/168, 13.10%). The mortality rate was highest for sunitinib-related ONJ (12/65, 18.46%), excluding missing data (n = 12), followed by sorafenib-related ONJ (2/13, 15.38%).

In ONJ-related ICSRs for ICIs, the highest number of ICSRs occurred in 2018. ICSRs on nivolumab were collected since 2017, and the highest number of ICSRs occurred in 2018. For pembrolizumab, ONJ AE reports were collected since 2018, and the highest number of ICSRs occurred in 2018, 2020, and 2022. In general, more males (62.22%) suffered ONJ AEs, and most of the cases were from North America and Europe. The median age of patients for all the ICI-related ICSRs was 63 years (IQR, 60–89 years; n = 36). More than half of the ICSRs related to male patients (62.2%). Unlike for TKIs, the most common indication for ICIs was lung cancer. The most common indication for nivolumab was renal cancer. For all the ICI-related ONJ documented in ICSRs (excluding missing data), the most common adverse outcome was other serious (17/39, 43.59%), and the most serious outcome was death/life-threatening condition (4/39, 10.26%), as shown in Tables 3–5.

**TABLE 3:** Demographic and clinical data of ONJ with interested TKIs.

	Axitinib (N = 24)		Cabozantinib (N = 10)		Lenvatinib (N = 40)		Pazopanib (N = 31)		Sunitinib (N = 77)		Sorafenib (N = 13)		Total (N = 195)	
Age median (range)	66	50-79	68	56-78	47	38-88	61	52-78	61	23-81	62	40-81	63	23-88
	N	%	N	%	N	%	N	%	N	%	N	%	N	%
Sex														
Females	5	20.83	0	0.00	15	37.50	10	32.26	18	23.38	4	30.77	52	26.67
Males	17	70.83	9	90.00	22	55.00	15	48.39	52	67.53	8	61.54	123	63.08
Missing	2	8.33	1	10.00	3	7.50	6	19.35	7	9.09	1	7.69	20	10.26
Reporter country														
North America	4	16.67	4	40.00	14	35.00	6	19.35	39	50.65	3	23.08	70	35.90
South America	2	8.33	0	0.00	0	0.00	0	0.00	3	3.90	0	0.00	5	2.56
Europe	9	37.50	4	40.00	9	22.50	17	54.84	26	33.77	6	46.15	71	36.41
Asia	8	33.33	1	10.00	15	37.50	5	16.13	9	11.69	4	30.77	42	21.54
Other	0	0.00	1	10.00	2	5.00	0	0.00	0	0.00	0	0.00	3	1.54
Missing	2	8.33	0	0.00	0	0.00	3	9.68	0	0.00	0	0.00	5	2.56
Outcome														
Death/life-threat	2	8.33	0	0.00	5	12.50	1	3.23	12	15.58	2	15.38	22	11.28
Disability	1	4.17	0	0.00	0	0.00	1	3.23	6	7.79	0	0.00	8	4.10
Hospitalization	2	8.33	5	50.00	18	45.00	6	19.35	17	22.08	2	15.38	50	25.64
Other serious	12	50.00	3	30.00	17	42.50	17	54.84	30	38.96	9	69.23	88	45.13
Missing	7	29.17	2	20.00	0	0.00	6	19.35	12	15.58	0	0.00	27	13.85
Indication														
Renal cancer	19	79.17	7	70.00	12	30.00	29	93.55	55	71.43	6	46.15	128	65.64
Hepatic cancer	0	0.00	0	0.00	5	12.50	0	0.00	0	0.00	1	7.69	6	3.08
Thyroid cancer	0	0.00	2	20.00	16	40.00	0	0.00	0	0.00	2	15.38	20	10.26
Other	5	20.83	1	10.00	7	17.50	2	6.45	22	28.57	4	30.77	41	21.03
Time to report														
2010	0	0.00	0	0.00	0	0.00	1	3.23	5	6.49	2	15.38	8	4.10
2011	0	0.00	0	0.00	0	0.00	0	0.00	17	22.08	1	7.69	18	9.23
2012	0	0.00	0	0.00	0	0.00	0	0.00	3	3.90	1	7.69	4	2.05
2013	0	0.00	0	0.00	0	0.00	4	12.90	0	0.00	3	23.08	7	3.59
2014	2	8.33	1	10.00	0	0.00	2	6.45	4	5.19	2	15.38	11	5.64
2015	1	4.17	1	10.00	0	0.00	6	19.35	8	10.39	0	0.00	16	8.21
2016	3	12.50	0	0.00	1	2.50	4	12.90	11	14.29	0	0.00	19	9.74
2017	3	12.50	1	10.00	1	2.50	3	9.68	6	7.79	0	0.00	14	7.18
2018	3	12.50	0	0.00	2	5.00	3	9.68	9	11.69	2	15.38	19	9.74
2019	0	0.00	2	20.00	7	17.50	2	6.45	6	7.79	1	7.69	18	9.23
2020	3	12.50	1	10.00	6	15.00	0	0.00	4	5.19	1	7.69	15	7.69
2021	2	8.33	1	10.00	10	25.00	0	0.00	3	3.90	0	0.00	16	8.21
2022	7	29.17	3	30.00	13	32.50	6	19.35	1	1.30	0	0.00	30	15.38

**TABLE 4:** Demographic and clinical data of ONJ with interested ICIs.

	Ipilimumab (N = 3)		Nivolumab (N = 30)		Pembrolizumab (N = 12)		Total (N = 45)	
Age median (range)	66	50-79	68	56-78	47	38-88	63	23-88
	N	%	N	%	N	%	N	%
Sex								
Females	1	33.33	3	10.00	8	66.67	12	26.67
Males	0	0.00	25	83.33	3	25.00	28	62.22
Missing	2	66.67	2	6.67	1	8.33	5	11.11
Reporter country								
North America	2	66.67	3	10.00	5	41.67	10	22.22
South America	0	0.00	0	0.00	0	0.00	0	0.00
Europe	1	33.33	17	56.67	3	25.00	21	46.67
Asia	0	0.00	10	33.33	4	33.33	14	31.11
Outcome								
Death/life-threat	0	0.00	3	10.00	1	8.33	4	8.89
Disability	0	0.00	1	3.33	0	0.00	1	2.22
Hospitalization	0	0.00	12	40.00	2	16.67	14	31.11
Other serious	0	0.00	12	40.00	5	41.67	17	37.78
Missing	3	100.00	2	6.67	4	33.33	6	13.33
Indication								
Renal cancer	0	0.00	12	40.00	2	16.67	14	31.11
Lung cancer	1	33.33	11	36.67	5	41.67	17	37.78
Malignant melanoma	0	0.00	4	13.33	1	8.33	5	11.11
Other	2	66.67	3	10.00	4	33.33	9	20.00
Time to report								
2017	0	0.00	3	10.00	0	0.00	3	6.67
2018	0	0.00	8	26.67	3	25.00	11	24.44
2019	2	66.67	4	13.33	1	8.33	7	15.56
2020	1	33.33	4	13.33	3	25.00	8	17.78
2021	0	0.00	4	13.33	2	16.67	6	13.33
2022	0	0.00	7	23.33	3	25.00	10	22.22

**TABLE 5:** Demographic and clinical data of ONJ with combined drugs.

	Cabozantinib + Nivolumab (N = 6)		Pembrolizumab + Lenvatinib (N = 4)		Pembrolizumab + Axitinib (N = 1)	
Age median (range)	58	43-65	69	57-81	57	57-57
	N	%	N	%	N	%
Sex						
Females	5	83.33	2	50.00	0	0.00
Males	1	16.67	2	50.00	1	100.00
Missing	0	0.00	0	0.00	0	0.00
Reporter country						
North America	0	0.00	1	25.00	0	0.00
Europe	6	100.00	1	25.00	1	100.00
Asia	0	0.00	2	50.00	0	0.00
Outcome						
Hospitalization	4	66.67	0	0.00	0	0.00
Other serious	1	16.67	4	100.00	1	100.00
Missing	1	16.67	0	0.00	0	0.00
Indication						
Renal cancer	6	100.00	1	25.00	1	100.00
Endometrial cancer	0	0.00	2	50.00	0	0.00
Other	0	0.00	1	25.00	0	0.00
Time to report						
2018	1	16.67	0	0.00	0	0.00
2019	0	0.00	0	0.00	0	0.00
2020	0	0.00	0	0.00	0	0.00
2021	1	16.67	0	0.00	0	0.00
2022	4	66.67	4	100.00	1	100.00

A total of 135 cases (ICIs: 20; TKIs: 115) were suitable for calculating the median time to onset value. We found that the median time to onset was 834 days for axitinib-related ICSRs, 853 days for cabozantinib-related ICSRs, 867 days for lenvatinib-related ICSRs, 877 days for pazopanib-related ICSRs, 921 days for sunitinib-related ICSRs, and 840 days for sorafenib-related ICSRs. Additionally, the overall median time to onset values for TKI-related ICSRs and ICI-related ICSRs were 840 days and 666 days, respectively.

### 3.3 Disproportionality analysis

For monotherapy, ONJ ICSRs were reported for all six TKIs (axitinib, cabozantinib, lenvatinib, pazopanib, sunitinib, and sorafenib). In particular, the majority of TKI-associated ONJ events were reported for sunitinib (39.5%), followed by lenvatinib (20.5%). Concurrently, based on the ROR criterion, sunitinib and lenvatinib were significantly associated with over-reporting of ONJ (ROR: 1.84, 95% confidence interval [CI]: 1.47–2.31; and ROR: 1.86, 95% confidence interval [CI]: 1.36–2.54, respectively). Among the four ICIs (avelumab, ipilimumab, nivolumab and pembrolizumab), no avelumab-related ONJ ICSRs were found. The majority of ICI-associated ONJ events were reported for nivolumab (66.7%), followed by nivolumab (26.7%). However, the four ICIs showed no significant association with over-reporting of ONJ.

For the six combination therapies (lenvatinib + everolimus, nivolumab + ipilimumab, pembrolizumab + axitinib, avelumab + axitinib, nivolumab + cabozantinib, pembrolizumab + lenvatinib), only the combinations of cabozantinib + nivolumab, pembrolizumab + lenvatinib, and pembrolizumab + axitinib had related ICSRs. Moreover, the majority of combination therapy-related ICSRs were reported for cabozantinib + nivolumab (54.5%), followed by pembrolizumab + lenvatinib (36.4%). However, with the criteria of Ω shrinkage, only the combination therapy of cabozantinib + nivolumab was significantly associated with over-reporting of ONJ (Ω 1.94, Ω025 1.30), as shown in Table 6.

**TABLE 6:** Associations of different interested drugs with ONJ.

Drugs	N	ROR (95%CI)	Ω (Ω025)
TKIs	195	1.31 (1.14-1.51)	
Axitinib	24	1.41 (0.94-2.10)	
Cabozantinib	10	1.24 (0.67-2.30)	
Lenvatinib	40	1.86 (1.36-2.54)	
Pazopanib	31	1.00 (0.70-1.42)	
Sunitinib	77	1.84 (1.47-2.31)	
Sorafenib	13	0.43 (0.25-0.75)	
ICIs	45	0.36 (0.27-0.48)	
Nivolumab	30	0.43 (0.30-0.62)	
Ipilimumab	3	0.22 (0.07-0.67)	
Pembrolizumab	12	0.29 (0.16-0.51)	
Cabozantinib + Nivolumab	6		1.94 (1.30)
Pembrolizumab + Lenvatinib	4		0.41 (-0.23)
Pembrolizumab + Axitinib	1		−0.48 (-1.12)

## 4 Discussion

To the authors’ knowledge, this is the first analysis that uses a pharmacovigilance approach to investigate the relationship between the use of TKIs and ICIs, either alone or in combination, and an increased risk of ONJ. Considering that TKIs and ICIs are widely used to treat kidney cancer, the finding of an association between ONJ occurrence and two monotherapy TKIs (sunitinib and lenvatinib) and one combination treatment (cabozantinib + nivolumab) is a significant finding. We also reviewed 25 cases of TKI-related ONJ and 4 cases of ICI-related ONJ that were reported in the literature.

In previous years, it was considered that patients with RCC had a lower susceptibility to ONJ than other solid tumors due to their often-limited survival time and short time period of NBP therapy. However, life expectancy in patients with metastatic RCC has nearly tripled since the introduction of targeted treatments (Heng et al., 2009), resulting in prolonged NBP exposure and an increased risk of ONJ.

The pathogenesis of medication-related osteonecrosis of the jaw has not been fully understood and may involve a number of factors in particular microenvironments. Multiple signalling pathways may play a role in the pathogenesis of medication-related osteonecrosis of the jaw. So far, it has been shown that TGF- β 1 signaling pathway plays an important role in the development of medication-related osteonecrosis of the jaw (Wehrhan et al., 2011; Kim et al., 2016; Lee et al., 2019). Some potential causes of ONJ associated with TKIs has been identified and supported by a number of published studies in recent years, such as TKIs might inhibit PDGFR’s effect on osteoclast and osteoblast (Abel Mahedi Mohamed et al., 2018; Patel et al., 2017; Viviano et al., 2017). However, in many cases, the risk of ONJ cannot be quantified or the underlying cause defined. Generally, TKI-related ONJ may occur spontaneously or post-surgically, and it more frequently occurs in the mandible. Moreover, the time of onset of ONJ after starting TKI use can vary (from weeks to 15 months) (Eguia et al., 2020). The pathogenesis of ICI-related ONJ is still unknown, but it may be related to reduced bone turnover due to changes in osteoclasts, inhibition of angiogenesis, infection, inflammation, and soft tissue toxicity (Marx, 2003).

In this work, both the pharmacovigilance results and case series indicated that the regimens most commonly associated with ONJ were sunitinib, lenvatinib, and nivolumab. The median age of patients in the TKI-related cases was 63 years (IQR, 56–70 years) in our pharmacovigilance analysis and 58 years (IQR, 51–62 years) for the cases of TKI-induced ONJ published in the case reports. The median age of patients in the ICI-related cases was 63 years (IQR, 60–89 years) in our pharmacovigilance analysis and 53.25 years (IQR, 46–62 years) for the cases of ICI-induced ONJ published in the case reports. A potential cause for the variation in the age of patients in the pharmacovigilance analysis compared to previous literature reports is the differences in sample size. Additionally, the mean time to onset of ONJ was twice as long as in the previous literature for both TKIs and ICIs.

In our pharmacovigilance analysis, drug-related ONJ events for all six TKIs were found, but in previous literature, only drug-related ONJ events were only reported for sunitinib, cabozantinib, and axitinib. Furthermore, there were far more cases reported in the pharmacovigilance analysis than in the literature due to the fact that patients examined in the previous literature had limited survival time, and the majority were not treated for long periods. Avelumab-related ONJ events were not reported either in FAERS or in prior literature. Our study identified a significant association between sunitinib, lenvatinib, and the combination of nivolumab + cabozantinib and ONJ, but these results were not reported in the previous literature. Additionally, although this identified association does not imply a biological causal relationship, Bozas and coworkers suggested a potential synergy between sunitinib and zoledronic acid in the induction of ONJ (Owosho et al., 2015).

Following the thorough study of ONJ, a variety of treatments have been proposed that may have some impact on the outcome of the disease. However, there are no clear guidelines for standardizing the selection of a therapeutic approach from a range of efficient alternatives. Based on a thorough study of medication-related osteonecrosis of the jaw, conservative treatment is the main therapy (He et al., 2020). In phases 0 and 1 of the disease, the main objective of medication is to prevent infection and control symptoms. As the disease progresses to phases 2 and 3, drugs such as teriparatide, pentoxifylline, and tocopherol may be used to delay the progression of the disease. If treatment with low-intensity laser or adjunctive therapy for more than 2 weeks shows no evident improvement, conservative surgical removal of the entire bone may be considered to minimize inflammation. Meanwhile, platelet-rich plasma and high-pressure oxygen can improve the success rate of operation. Once the bone infection is under control, it is necessary to consider an adjacent flap (e.g., buccal fat pad or improved submental island flap) in order to repair soft tissue defects. In the third stage, if there is no effective conservative therapy, radical surgery may be performed to improve the patient’s quality of life. Indeed, the reconstruction of the dead space after the removal of the damaged bone can be carried out by means of free flap repair (Abel Mahedi Mohamed et al., 2018).

To obtain higher-quality evidence in this area of research, attention should be paid to ONJ caused by TKIs and ICIs in randomized controlled studies. Additionally, more data are required to evaluate the incidence of and risk factors associated with ONJ development. Furthermore, clarification of the mechanisms of these AEs at the molecular and cellular levels is needed to develop more efficacious drug therapies. Finally, there is a need for evidence-based guidance to manage ONJ in patients with renal cancer.

Our study had three main limitations (Guo et al., 2023). Firstly, data mining does not compensate for the inherent shortcomings of the FAERS, such as false, incomplete, and inaccurate reporting, all of which may result in bias. The absence of laboratory values and complete medical records (including comprehensive information on concomitant medications and comorbidities) may have contributed to errors in our analysis. Secondly, only qualitative research could be used in our study due to the intrinsic characteristics of the FAERS. Therefore, it was not possible to quantify the prevalence of ONJ as AEs compared with overall AEs due to TKI and ICI use. Thirdly, even if pharmacovigilance databases suggest a significant correlation between a targeted drug and a target AE, this does not imply causality. The FAERS has limitations in terms of its inclusion of genetic factors, but it does indicate certain key features of ONJ in response to TKI and ICI use, such as the timing, spectrum, and clinical manifestations of ONJ, which may provide useful insights for the development of further well-designed research.

## 5 Conclusion

Overall, our findings support the assumption that ONJ AEs are a safety concern for the use of TKIs and ICIs (which are used in the treatment of renal cell carcinoma). Therefore, patients with renal cancer who intend to receive sunitinib or lenvatinib or a combination of nivolumab + cabozantinib in a non-emergency situation should have their oral care assessed before initiating therapy to ensure appropriate dental care. Dental monitoring should be performed according to a routine regimen (e.g., every 6 months after initiation of therapy), but there is currently no detailed schedule for disease follow-up, and proper management of ONJ during anticancer therapy should not be overlooked. Furthermore, a large prospective population-based study is required to determine the true incidence of AEs and to fully clarify the risk factors for certain AEs, as this would support appropriate risk management.

## Data Availability

The original contributions presented in the study are included in the article/supplementary material, further inquiries can be directed to the corresponding authors.
